# Diagnosis of Partial Body Radiation Exposure in Mice Using Peripheral Blood Gene Expression Profiles

**DOI:** 10.1371/journal.pone.0011535

**Published:** 2010-07-12

**Authors:** Sarah K. Meadows, Holly K. Dressman, Pamela Daher, Heather Himburg, J. Lauren Russell, Phuong Doan, Nelson J. Chao, Joseph Lucas, Joseph R. Nevins, John P. Chute

**Affiliations:** 1 Division of Cellular Therapy, Department of Medicine, Duke University, Durham, North Carolina, United States of America; 2 Institute for Genome Sciences and Policy, Duke University, Durham, North Carolina, United States of America; 3 Department of Pharmacology and Cancer Biology, Duke University, Durham, North Carolina, United States of America; Dresden University of Technology, Germany

## Abstract

In the event of a terrorist-mediated attack in the United States using radiological or improvised nuclear weapons, it is expected that hundreds of thousands of people could be exposed to life-threatening levels of ionizing radiation. We have recently shown that genome-wide expression analysis of the peripheral blood (PB) can generate gene expression profiles that can predict radiation exposure and distinguish the dose level of exposure following total body irradiation (TBI). However, in the event a radiation-mass casualty scenario, many victims will have heterogeneous exposure due to partial shielding and it is unknown whether PB gene expression profiles would be useful in predicting the status of partially irradiated individuals. Here, we identified gene expression profiles in the PB that were characteristic of anterior hemibody-, posterior hemibody- and single limb-irradiation at 0.5 Gy, 2 Gy and 10 Gy in C57Bl6 mice. These PB signatures predicted the radiation status of partially irradiated mice with a high level of accuracy (range 79–100%) compared to non-irradiated mice. Interestingly, PB signatures of partial body irradiation were poorly predictive of radiation status by site of injury (range 16–43%), suggesting that the PB molecular response to partial body irradiation was anatomic site specific. Importantly, PB gene signatures generated from TBI-treated mice failed completely to predict the radiation status of partially irradiated animals or non-irradiated controls. These data demonstrate that partial body irradiation, even to a single limb, generates a characteristic PB signature of radiation injury and thus may necessitate the use of multiple signatures, both partial body and total body, to accurately assess the status of an individual exposed to radiation.

## Introduction

In the event of a terrorist-driven detonation of an improvised nuclear device (IND) in a populated U.S. city, it is expected that hundreds of thousands of people could be exposed to ionizing radiation, with even larger numbers fearful that they have been exposed [1]–[4]. Over the past 5 years, U.S. federal, state and local governments and leading medical societies have spearheaded efforts to organize the medical response to such an event and highly considered, well-conceived therapeutic guidelines have been made publicly available for health care providers to have “just in time' algorithms as to how to treat radiation victims should an event occur [5], [6]. However, the successful implementation of any large scale medical response for a mass casualty radiation event will depend upon the availability and utility of diagnostic tests to determine radiation exposure status and dose of exposure among victims and the availability of therapeutics that can be administered to mitigate radiation damage to vital organ systems [2]–[4], [7], [8].

We have applied genome-wide analytical methods and high-throughput computational tools to determine whether “signatures” of radiation injury can be identified in the peripheral blood (PB) of mice and humans following exposure to several dose levels of gamma irradiation [9]. Utilizing a binary regression analysis, patterns of gene expression (50–100 genes) were identified in the PB of mice that were capable of predicting radiation status and distinguishing the dose level of exposure between non-irradiated, 0.5 Gy-, 2 Gy- and 10 Gy-irradiated animals with accuracy of 96% [9]. We subsequently applied this same approach to predicting the radiation status of humans who received total body irradiation (TBI) prior to stem cell transplantation as compared to non-irradiated patients and healthy human controls and found that a PB signature of 25 genes was capable of predicting the radiation status of humans with an overall accuracy of 95% [10]. Taken together, these studies confirmed the power of PB gene expression profiles or “metagenes” to predict the radiation status of people and provided the basis for our current effort to develop a rapid, high throughput biodosimetry assay for application in a radiation mass casualty scenario.

While these studies have clearly identified PB metagenes that can predict radiation status and dose of exposure after total body irradiation (TBI), an important refinement to these signatures would be incorporation of analysis of partially-exposed individuals; this is particularly important in the development of an biodosimetry assay for acute radiation injury since it is expected that a large percentage of radiation victims in a mass casualty scenario will have heterogeneous exposures due to partial shielding [11]–[13]. Here, we identify PB gene expression profiles of partial body irradiation that can predict the radiation status of partially irradiated animals with a high degree of accuracy. We also show that such PB signatures can potentially distinguish the anatomic site of radiation exposure and that PB signatures generated from TBI-treated animals fail to predict the radiation status of partially irradiated animals. An algorithm which incorporates TBI- and partial body-signatures can allow rapid determination of the health of individuals in a mass casualty radiation event.

## Methods

### Murine irradiation study

Twelve week old female C57Bl6 mice (Jackson Laboratory, Bar Harbor, ME) were housed at the Duke Cancer Center Isolation Facility and all protocols in this study were approved by the Duke University Animal Care and Use Committee (Protocol Number A037-10-02). Six to seven mice/group were treated with partial body irradiation to either the anterior hemibody (AH), posterior hemibody (PH), or hind limb (HL) regions with an X-ray source at doses of 0.5, 2, or 10 Gy using a filter of 0.1 mm Cu and 2.5 mm Al. After dosimetry studies were performed to assess absorbed dose in the body and in the hind limb of the animal, anterior and posterior regions of the mice were irradiated at an average of 1.49 Gy/min and the hind limbs at an average of 1.25 Gy/min. Six hours post-irradiation, approximately 500 ul peripheral blood was collected by cardiac bleed from both irradiated and control mice. PB mononuclear cells (PB MNCs) were isolated by Lymphoprep density gradient centrifugation (Axis-Shield PoC AS, Oslo, Norway) and total RNA was extracted with a Qiagen RNEasy Mini Kit (Qiagen Inc., Valencia, CA) as previously described [9], [10]. RNA quality was assayed using an Agilent Bioanalyzer 2100 (Agilent Technologies, Inc., Palo Alto, CA).

### DNA Microarrays

Mouse and human oligonucleotide arrays were printed at the Duke Microarray Facility using Operon's Mouse Genome Oligo sets (version 4.0). Operon's Mouse Genome Oligo set (version 4.0) (https://www.operon.com/arrays/oligosets_mouse.php) contains 35,852 oligonucleotide probes representing 25,000 genes and approximately 38,000 transcripts. In comparing to previously published total body irradiation dataset [9], [10], Operon provided a map that matched the probes from both versions and only these were used in the analysis.

### RNA and Microarray Probe Preparation and Hybridization

Briefly, MNCs were pelleted, and total RNA was isolated using the RNeasy mini spin column as previously described [10]. Total RNA from each sample and the universal reference RNA (Universal Mouse Reference RNA, Stratagene, http://www.stratagene.com) were amplified and used in probe preparation as previously described [9]. The sample was labeled with Cy5 and the mouse reference was labeled with Cy3. The reference RNA allows for the signal for each gene to be normalized to its own unique factor allowing comparisons of gene expression across multiple samples. This serves as a normalization control for two-color microarrays and an internal standardization for the arrays. Amplification, probe preparation and hybridization protocols were performed as previously described [9] and multiple replicates were examined in each condition. Detailed protocols are available on the Duke Microarray Facility web site (http://microarray.genome.duke.edu/services/spotted-arrays/protocols).

### Data Processing and Statistical Analysis

Genespring GX 7.3 (Agilent Technologies) was used to perform Lowess normalization of the data and then the data was filtered in which spots whose signal intensities below 70 in either the Cy3 or Cy5 channel were removed. To then account for missing values, PAM software (http://www-stat.stanford.edu/tibs/PAM/) was used to impute missing values. *k*-nearest neighbor was used where missing values were imputed using a *k*-nearest neighbor average in gene space.

Gene expression profiles of dose response for anterior, posterior and hind limb irradiation were used in three different statistical analyses: 1) a supervised analysis using binary regression methodologies as described previously [9], [10], 2) an unsupervised “latent factor” analysis described in [14] and exemplified in [15]–[17], and 3) a standard supervised analysis utilizing t-tests and correction for multiple testing. The additional analyses were performed to validate the poor performance of the supervised binary predictor when the model was built using total body radiation exposure and used to predict partial body radiation exposure.

Prediction analysis of the expression data based on the supervised binary regression analysis was performed using MATLAB software as previously described [9]. When predicting levels of radiation exposure, gene selection and identification is based on training the data and finding those genes most highly correlated to radiation exposure. Each signature summarizes its constituent genes as a single expression profile and is here derived as the first principal component of that set of genes (the factor corresponding to the largest singular value), as determined by a singular value decomposition. Given a training set of expression vectors (of values across metagenes) representing two biological states, a binary probit regression model is estimated using Bayesian methods. Bayesian fitting of binary probit regression models to the training data permits an assessment of the relevance of the metagene signatures in sample classification. The regression models are assigned binary regression weights which map metagenes to probabilities of radiation exposure. To internally validate the predictive capacity of the metagene profiles, we performed leave-one-out cross validation studies as we have previously described [9]. A leave one out cross validation involves removing one sample from the dataset, using the remaining samples to develop the model, and then predicting the status of the held out sample. This is then repeated for each sample in the dataset. We have utilized this approach as previously described [9], [10]. A ROC curve analysis was used to define a cut-off for sensitivity and specificity in the predictive models of radiation. All microarray data files will be submitted and available at the gene expression omnibus (GEO) website.

Analysis based on unsupervised factor models was carried out with publicly available software as previously described [18]. The total body radiation exposure was modeled using twenty three latent factors without regard to the radiation exposure dosage (unsupervised). These factors were then used to build a binary predictor of radiation exposure. Performance of this classifier on the training data set was perfect, indicating a clear, strong response in the peripheral blood to total body radiation exposure. These same factors were then projected onto the data from partial body exposure, as described in [16]. The “exposure” model was then tested for performance on the partial body radiation data. Finally, all genes were tested for association with total body and partial body radiation exposure (ANOVA) and p-values signifying the strength of association were generated.

### Murine cell subset analyses

Mice were irradiated at the doses described previously (n = 2 non-irradiated; n = 3 for all other doses). At 6 hours post irradiation, PB MNCs were isolated and stained for flow cytometry using rat anti mouse APC-Ter-119, APC-B220, PE-Mac-1, PE-Gr-1, and FITC-Thy1.2 antibodies (Becton Dickinson, BD). Cell subsets were analyzed as a percentage of the live cell population. For the survival analysis, mice were irradiated with the X-ray source at 10 Gy to either AH (n = 10) or PH (n = 10) as described above. Mice were followed for 60 days post-irradiation to assess differences in survival.

## Results

### PB signature of anterior hemibody (AH) irradiation

**Figure 1:**
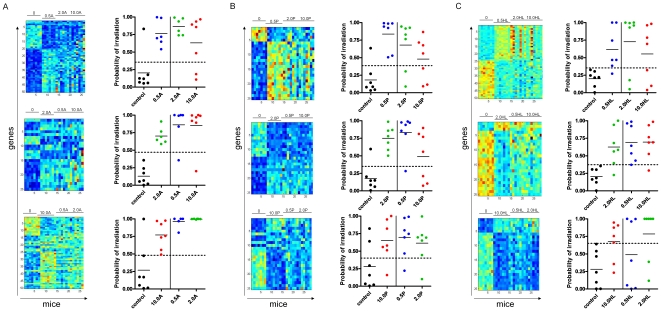
Gene expression profiles that reflect partial body irradiation. At left, gene expression patterns in the peripheral blood of mice are shown which were selected for predicting irradiation of (A) anterior hemibody (AH), (B) posterior hemibody (PH) irradiation, and (C) hind limb (HL) irradiation versus no irradiation at 0.5 Gy (top), 2 Gy (middle) and 10 Gy exposure (bottom). Each column represents a sample from an individual experiment, and each row represents a gene. High expression is depicted as red and low expression is depicted as blue and the range of expression is 0.06 to 210. At right, leave one-out-cross validation analyses of the classification probabilities using the binary regression model (see Methods) of control vs. 0.5 Gy, control vs. 2 Gy, and control vs. 10 Gy are shown. Each dot represents a PB sample from an individual mouse. These analyses demonstrate that the signatures of (A) AH irradiation for 0.5 Gy, 2 Gy and 10 Gy were highly accurate at predicting the status of AH-irradiated from non-irradiated mice (92%, 100% and 93% accuracy for 0.5, 2, and 10 Gy, respectively), but demonstrated less accuracy in distinguishing dose levels. (B) The signatures of PH irradiation were highly accurate at predicting radiation status versus control mice but showed less accuracy at distinguishing radiation dose levels. (C) The signatures of HL irradiation were highly accurate at predicting radiation status versus control mice but showed less accuracy at distinguishing radiation dose levels.

We first sought to determine if irradiation of one-half of the body could produce a PB gene expression response that was characteristic of that level of radiation exposure and whether irradiation of the AH (head to T12), PH (below T12) or HL (single hind limb) produced unique PB gene expression profiles. Of note, irradiation to the AH encompasses the spleen, whereas irradiation to the PH encompasses the pelvis and both femurs. Twelve week old C57Bl6 female mice (n = 6–7 per group) were irradiated with single fractions of 0.5 Gy, 2 Gy or 10 Gy and PB was collected at 6 hours post-irradiation for analysis. We chose these dose levels since they reflect medically distinct exposure levels which require different levels of intervention (e.g. 10 Gy is 100% lethal) [1]–[4], [9]. In order to determine if there was structure evident in the gene expression response to AH irradiation, we performed a supervised binary regression analysis of PB samples from mice irradiated with 0.5, 2, and 10 Gy AH irradiation. A pattern of gene expression could be identified that effectively distinguished mice irradiated to AH compared to non-irradiated mice (Figure 1A). In order to validate that these patterns did indeed represent genes reflecting exposure to AH irradiation, we performed a leave-one-out cross validation analysis to assess the ability of the pattern to predict the radiation status of unknown PB samples. The results demonstrate that the pattern selected for distinguishing AH-irradiated animals from non-irradiated controls does indeed have the capacity to predict the radiation status of PB samples from irradiated and non-irradiated mice with an accuracy of 92%, 100% and 93% for the 0.5, 2, and 10 Gy signatures (Figure 1A). We conclude that irradiation of the AH produces a PB gene signature of radiation that reflects radiation status and can potentially be used to predict radiation status.

### PB signature of PH irradiation

We also irradiated a group of mice (n = 7 per dose level) to the PH and identified PB gene expression profiles for each dose level that reflected PH irradiation (Figure 1B). A leave-one-out cross validation analysis was performed and demonstrated that the pattern of gene expression reflective of PH irradiation was capable of predicting the radiation status of PH-irradiated mice versus non-irradiated control mice with an accuracy of 93%, 93% and 79% for 0.5 Gy, 2 Gy and 10 Gy dose levels (Figure 1B).

### PB signature of HL irradiation

Since we were able to identify PB signatures of radiation that reflected AH and PH irradiation, we sought to determine if irradiation to a single limb produced a PB gene expression profile that could comparably predict radiation status versus non-irradiated controls. Interestingly, we identified patterns of PB gene expression that appeared to distinguish HL-irradiated mice from non-irradiated control mice using a binary regression analysis (Figure 1C). When we applied a leave-one-out cross validation analysis, we found that the PB signature of 0.5, 2, and 10 Gy were surprisingly accurate at predicting the radiation status of mice irradiated to a single hind limb as compared to non-irradiated controls (Accuracies: 93%, 93% and 86%, respectively; Figure 1C). Taken together, these results demonstrated the sensitivity of PB genome wide expression analysis toward detecting radiation exposure even in the setting of a partial body exposure to less than 25% of the body surface area.

### Partial body signatures poorly discriminate dose levels

Since the health effects of radiation exposure are a direct function of the dose level of exposure, it would be practically important for any bioassay for radiation exposure to have the capacity to discriminate different dose levels. In our prior studies of TBI exposure in mice, we identified PB gene expression profiles which were capable of discriminating dose levels of 0.5, 2, and 10 Gy TBI [9], [10]. Such discrimination is important since 0.5 Gy exposure causes no acute health effects, whereas 2 Gy is myelosuppressive and immunosuppressive and 10 Gy is a lethal exposure. In the current study, we found that the gene expression profiles of AH-, PH- and HL-irradiation were not accurate at discriminating one dose level of irradiation from another. For example, the overall accuracy of the AH 0.5 Gy signature at distinguishing 0.5 Gy-irradiated samples from non-irradiated, 2 Gy-irradiated or 10 Gy-irradiated was 54% overall (Figure 1A). Similarly, the PH 2 Gy signature demonstrated an overall accuracy of 61% in distinguishing that dose level versus the other dose levels of PH exposure (Figure 1). None of the PB profiles within the AH, PH or HL conditions demonstrated an accuracy greater than 60% in distinguishing dose level (Figure 1). Consistent with these results, we found only 6 genes in common between 0.5, 2, and 10 Gy gene expression profiles within the AH condition: *aryl hydrocarbon receptor (AHR), neuropsin (Prss19), R-spondin, lectin-galactose binding-soluble 3 (Lgals3), NTF2-related export protein*, and *nuclear factor-IL3 regulated*; no genes were in common between the 3 dose levels in the PH condition and *Pscd3* and *Slc41a3* were in common at all dose levels in the HL group (Table 1 and Table S1). Taken together, these data indicate that partial body radiation exposure induces distinct molecular responses in the PB as a function of dose level.

**Table 1:** Overlap genes between 0.5, 2.0 and 10 Gy dose levels in partially irradiated mice.

Operon OligoID	Gene Symbol	RefSeq	GenBank	Description
**Anterior 0.5 Gy, 2.0 Gy, and 10.0 Gy**				
M200001752	Ahr	NM_013464		aryl-hydrocarbon receptor
M200002206	Prss19	NM_008940	D30785	protease, serine, 19 (neuropsin)
M200013923	Rspondin	NM_138683	AB016768	thrombospondin type 1 domain containing gene
M300021033	Lgals3	NM_010705		lectin, galactose binding, soluble 3
M400001965	Nxt1	NM_019761	AA915380	NTF2-related export protein 1
M400005620	Nfil3	NM_017373		nuclear factor, interleukin 3, regulated
**Posterior 0.5 Gy, 2.0 Gy, and 10.0 Gy**				
NONE				
**Hind Limb 0.5 Gy, 2.0 Gy, and 10.0 Gy**				
M200003725	Pscd3	NM_011182	BC035296	pleckstrin homology, Sec7 and coiled-coil domains 3
M200007299	Slc41a3	XM_132686		PREDICTED: solute carrier family 41, member 3
M400014572				

### Partial body signatures are unique to the anatomic site that is irradiated

**Figure 2:**
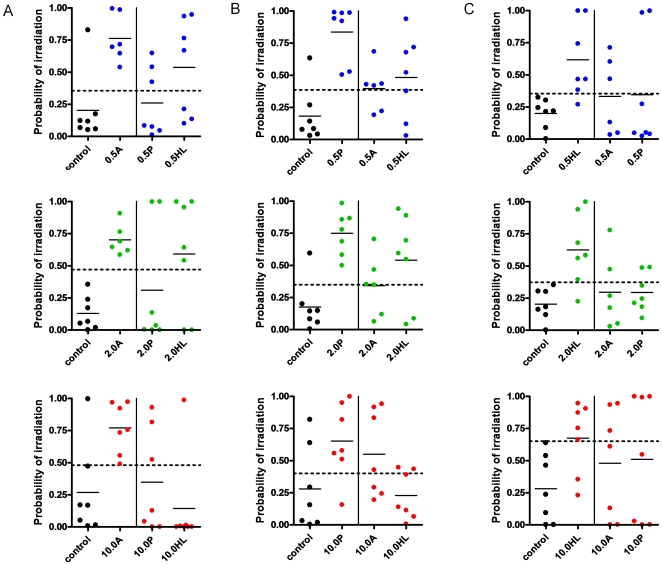
Gene expression profiles of partial body irradiation poorly predict the status of mice irradiated to disparate anatomic sites. A single variable plot is shown of the leave one out cross validation analysis performed in which the gene expression profiles of (A) AH irradiation were utilized to predict the status of mice irradiated to PH or HL at the identical dose levels (0.5 Gy, top; 2 Gy, middle; 10 Gy, bottom). (B) PB signatures of PH irradiation were tested against mice irradiated to AH and HL at 0.5 Gy (top), 2 Gy (middle) and 10 Gy (bottom). (C) PB signatures of HL were tested against mice irradiated to AH and PH at 0.5 Gy (top), 2 Gy (middle) and 10 Gy (bottom). Positive prediction of radiation status is defined by plotting of the sample above the ROC curve-defined cutoff (dotted line). Each dot represents a PB sample from an individual mouse.

We next sought to determine if PB signatures of partial body irradiation were capable of predicting the status of other partially irradiated mice in which different parts of the body had been irradiated. The predictors of partial irradiation to AH, PH and HL (n = 25-50 genes from Figure 1) were utilized to predict radiation status by anatomic site. Surprisingly, the PB signatures of partial body irradiation demonstrated low accuracy in predicting the radiation status of other mice irradiated at the same dose level to other parts of the body (Figure 2). For example, the PB signature of 10 Gy AH failed to predict the status of 57% and 86% the PB samples from mice irradiated with 10 Gy to the PH or HL, respectively. This lack of accuracy in predicting radiation status of PB samples from partially irradiated mice was irrespective of dose level and anatomic location; for example, the PB signature of 2 Gy HL exposure failed to predict the radiation status of 66% and 71% of the PB samples from mice irradiated with 2 Gy to the AH or PH, respectively. Consistent with these results, we found little overlap in genes represented within the AH, PH or HL radiation groups at any dose level and only 1 gene which overlapped between all 3 conditions (*RIKEN cDNA 6330579B17*) at the 10 Gy dose level (Table S2). Taken together, these data demonstrate that ionizing radiation induces distinct PB molecular responses as a function of the anatomic site of exposure, rather than a redundant molecular response based upon the percentage of body surface area that is irradiated.

### PB signatures of TBI fail to predict the status of partially irradiated mice

**Figure 3:**
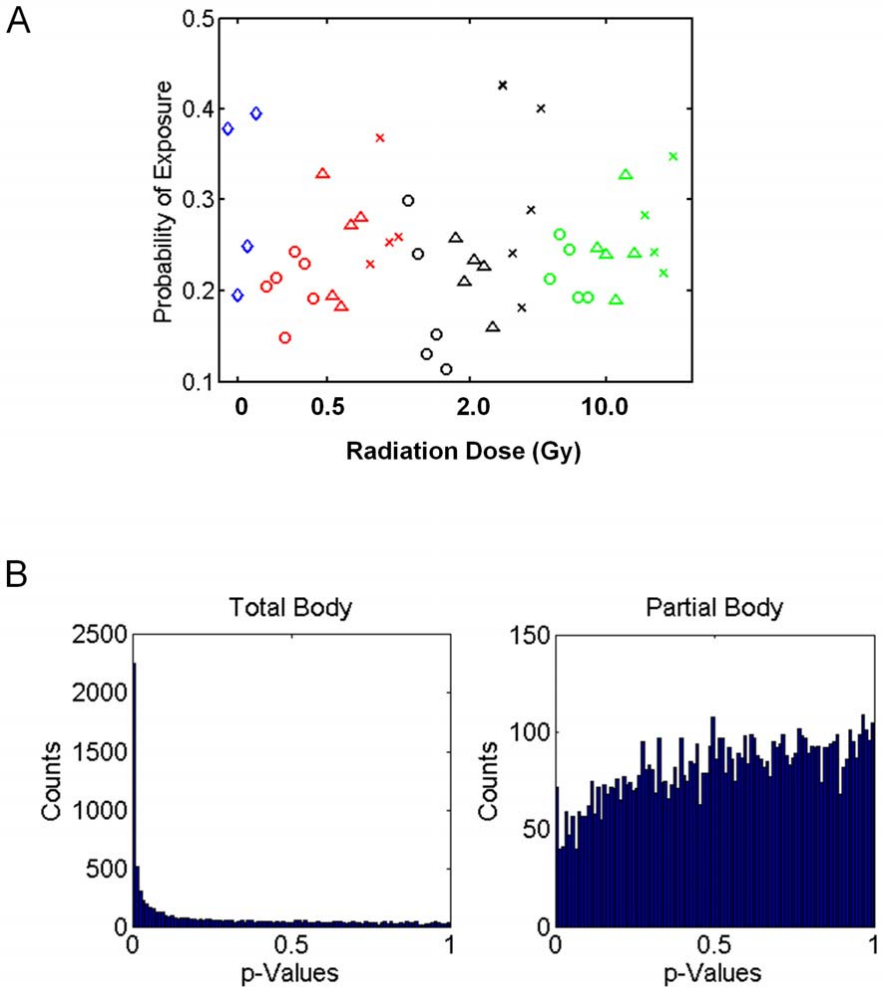
Prediction of radiation status of partially irradiated mice based upon the TBI gene expression profiles. (A) Diamonds represent control (non irradiated) samples, circles are exposure to AH, triangles are exposure to PH and Xs represent exposure to HL. Different dose levels were tested (blue = non-irradiated, red = 0.5 Gy, black = 2 Gy, green = 10 Gy). The predictor built with the TBI samples shows no capacity to predict the radiation status of partially irradiated mice. (B) Histogram showing association of *P* values for association of genes with radiation exposure. A large increase in the number of small *P* values close to zero is observed compared to uniform distribution in the total body irradiation group (left). No trend is evident in *P* values in the partial body irradiated group of genes.

Since PB signatures of radiation injury developed from TBI-patients are currently being developed as biodosimetry assays for the screening of radiation mass casualties [9], [10], [13], [19], we sought to determine if the PB signatures generated from TBI-mice can accurately discriminate PB samples from partially irradiated mice. For this analysis, predictors of 0.5, 2, and 10 Gy developed from the PB of TBI-mice were tested against PB samples from mice exposed to 0.5, 2, or 10 Gy to AH, PH or HL. Interestingly, we found that none of the predictors of 0.5, 2, or 10 Gy irradiation generated from TBI-mice were able to predict the radiation status of partially irradiated mice at the identical dose levels from the AH, PH or HL groups (Figure 3A). Specifically, PB signatures built from TBI-mice were unable to distinguish partially irradiated mice from non-irradiated controls and could not discriminate dose levels in any animals. These results demonstrate that the total body model performs poorly in attempting to predict the radiation status of partially irradiated animals.

As an alternative approach to compare the PB gene expression profile from TBI versus partial body irradiation, all genes were tested for association with total body and partial body radiation exposure (ANOVA) and p-values signifying the strength of association were generated (Figure 3B). In order to test for the presence of genes that are strongly associated with radiation exposure, we perform 8151 independent t-tests. We found that there were 53 genes in the total body exposure group that showed significant differential expression even after Bonferroni correction for multiple hypotheses (*P*<.01/8151). In contrast, there was nearly uniform distribution of p-values generated from the partial body radiation, suggesting a much smaller response. This analysis does not preclude a groups of genes, each having a small response, from being used to build predictors, but does suggest that there are no strong single gene predictors for this phenotype. At the same time, there are none from partial body group which pass this test, and in addition, the smallest p-value in this group is more than an order of magnitude too large to qualify as significant. Taken together, these results indicate that PB signatures of radiation generated from TBI-recipients are unlikely to accurately predict radiation status in partially irradiated individuals.

As a corollary to the predictive analysis, we examined the genes represented within the PB signatures of 0.5, 2, and 10 Gy TBI versus the signatures of AH, PH and HL exposure at the same dose levels (Table S3). We found no more than 3 genes which overlapped between the TBI signatures and the partial body irradiation signatures at any dose level. For example, the PB signature of 10 Gy exposure in AH-treated mice had no genes in common with the PB signature of 10 Gy TBI-mice and only 2 genes (*Cdkn1a* and *Dcxr*) were found to be in common between the 10 Gy TBI signature and the 10 Gy signature from PH-treated mice (Table S3). Taken together, these results confirmed that partial body irradiation produces a wholly distinct molecular response in the PB compared to TBI at the same dose levels. As complementary evidence that the biologic response to partial body irradiation is distinct from TBI, we also found that adult C57Bl6 mice (n = 10/group) irradiated with 10 Gy to AH or PH had 100% survival through 60 days (data not shown), whereas10 Gy TBI is 100% lethal by day 30 in C57Bl6 mice [20].

### PB cell content differs following TBI versus partial body irradiation

**Figure 4:**
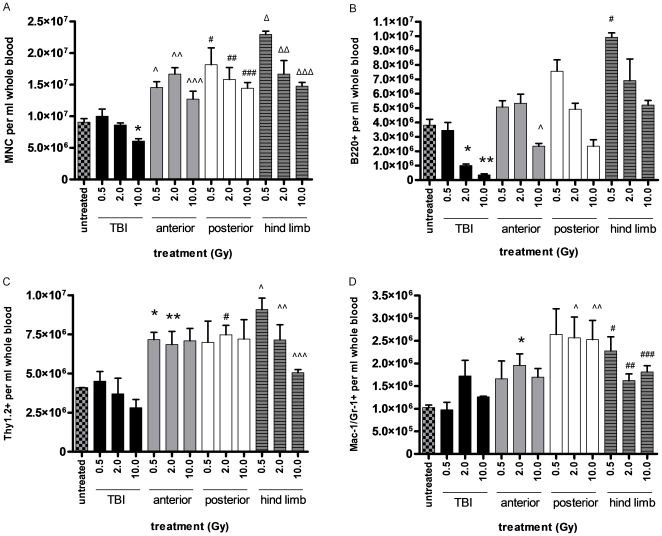
Partial body irradiation and TBI cause significantly different changes in PB cell distributions. (A) The mean numbers of PB MNCs are shown in non-irradiated (untreated) mice versus mice irradiated with TBI, AH, PH or HL irradiation. *P = 0.02 for comparison with non-irradiated mice; ∧ *P* = 0.007, ∧∧*P* = 0.004, ∧∧∧*P* = 0.02 for comparison with non-irradiated mice; ^#^ *P* = 0.03, ^##^ *P* = 0.03, ^###^ *P* = 0.007 for comparison with non-irradiated mice; ^Δ^ *P* = 0.0005, ^ΔΔ^ *P* = 0.03, ^ΔΔΔ^ *P* = 0.004 for comparison with non-irradiated mice. (B) TBI causes a significant decrease in PB B cells (B220^+^) at increasing dose levels. *P = 0.04, **P = 0.03; AH and PH irradiation decrease PB B cells at 10 Gy, ∧*P* = 0.03; HL irradiation increases PB B cell content at all doses, ^#^ *P* = 0.003. (C) TBI causes a modest decrease in PB T cells (Thy 1.2^+^) at 10 Gy; AH, PH and HL irradiation uniformly cause an increase in PB T cells compared to non-irradiated controls, *P = 0.01, **P = 0.04, ^#^ *P* = 0.01, ∧*P* = 0.01, ∧∧*P* = 0.04, ∧∧∧*P* = 0.01. (D) TBI causes no significant change in PB myeloid (Mac-1^+^) cells; mice irradiated to AH, PH or HL demonstrated an increase in PB myeloid cells at 6 hours, *P = 0.03, ∧*P* = 0.04, ∧∧*P* = 0.03, ^#^ *P* = 0.02, ^##^ *P* = 0.02, ^###^ *P* = 0.009.

Since partial body irradiation produced significantly different PB gene expression profiles compared to TBI, we analyzed PB from partially irradiated versus TBI-mice to determine if changes in PB cell content contributed to these differences. TBI caused a 33% decrease in PB MNCs within 6 hours of exposure (Figure 4), but partial body exposures caused an increase in PB MNCs compared to non-irradiated mice (Figure 4). TBI caused a modest increase in PB Mac-1^+^ myeloid cells and a modest decrease in Thy1.2^+^ T cells but both populations doubled in the PB following partial body irradiation to AH or PH. B220^+^ B lymphocytes decreased by >10-fold in the PB in response to TBI and were predominantly not affected by partial body irradiation. Taken together, these results suggest a model in which TBI causes a rapid and significant shift in the proportion of circulating PB cells which contribute to the PB gene expression profile (myeloid and T cells >>>> B cells) compared to partially irradiated mice. These differences between PB cell content in TBI- and partial body irradiated-mice may be explained, in part, by the generalized mobilization of hematopoietic cell subsets which occurs following partial body irradiation [21]. Since AH irradiation spares both femurs and PH irradiation spares the hematopoietic spleen, it is not surprising that mice irradiated to AH or PH would sustain PB cell counts whereas TBI-treated mice would not. These differences in PB cell content may also reflect the capacity for BM progenitor cells to mobilize into the PB in response to injury at distant anatomic sites [21], [22].

## Discussion

Gene expression profiles of solid tumors have been successfully applied to predict patient prognosis and the responsiveness of various cancers to different chemotherapies [23]–[26]. Peripheral blood (PB) gene expression profiles have also been applied to develop signatures of autoimmune diseases, stroke, bacterial and viral infections [27]–[31]. An important additional application of gene expression profiling would be to facilitate detection of exposure to environmental hazards, such as ionizing radiation and organic compounds (e.g. benzene). Exposure to such environmental hazards increases the longitudinal risk for hematologic diseases as well as the development of cancer over time [32], [33]. For example, repetitive CT scans as commonly performed in the follow up of young patients with a history of cancer, deliver significant radiation exposure which confers an increased risk of cancer development over time [34]. Similarly, repetitive occupational exposure to radiation, as occurs amongst interventional cardiologists and radiology technicians, may increase the lifetime risk of cancer [35], [36]. However, no test exists to measure the level of absorbed radiation dose (biodosimetry) or quantify the risk of such radiation exposure toward the development of cancer. Such concerns are magnified when considered in the context of the current well-articulated objective of terrorists to use radiological or improvised nuclear weapons to attack the United States; in the latter scenario, detonation of a “dirty bomb” or improvised nuclear device (IND) in a U.S. city could cause radiation injury to hundreds of thousands of individuals at one time.

We have sought to develop a PB assay for ionizing radiation exposure using gene expression profiles. Recently, we demonstrated that PB signatures of TBI exposure were capable of predicting both radiation status and dose level of exposure in mice with 96% accuracy [9]. We subsequently showed that a PB signature of as few as 25 genes developed in human patients exposed to TBI was capable of predicting the radiation status of irradiated and healthy individuals with an overall accuracy of 94% [10]. However, in the event of a radiological or nuclear detonation, it can be expected that a large percentage of exposed victims will have heterogeneous radiation exposure as a function of partial shielding [11]–[13]; therefore, PB signatures of TBI may not be predictive or applicable to diagnose radiation exposure in people who have suffered only partial body irradiation. Here, we found that irradiation of 50% of the body surface area (anterior or posterior) in mice with 0.5–10 Gy produced PB patterns of gene expression that were characteristic of these exposures. Interestingly, radiation exposure to a single hind limb also produced characteristic PB signatures in mice, suggesting that genome-wide analysis of the PB is quite sensitive to detect radiation exposure to a relatively small portion of the body surface area. We also demonstrate that such PB signatures of partial body irradiation are capable of predicting the radiation status of unknown PB samples from mice with an accuracy of 79–100%. However, the PB signatures of hemi-body or single limb irradiation were incapable of distinguishing the dose level of exposure between 0.5, 2, and 10 Gy. This is in sharp contrast to our prior observation that TBI exposure produced PB signatures of 0.5, 2, and 10 Gy which were highly accurate (96%) at predicting the dose level of exposure in mice [9]. We also found few genes in common between the predictors of 0.5, 2, or 10 Gy within any of the partial body irradiation conditions. One possible explanation for the inability of partial body irradiation signatures to discriminate dose levels accurately is that partial body irradiation produces a weaker molecular signal in the PB compared to TBI. It is likely that the gene expression profile of irradiation is muted in partially irradiated mice by the contribution of circulating, non-irradiated hematopoietic cells.

Despite the fact that we were able to identify PB signatures of partial body exposure that predicted radiation status within the AH, PH and HL exposure groups, we also found that the PB signatures of partial body irradiation failed to predict radiation status based upon site of exposure. For example, the PB signature of 2 Gy AH irradiation failed to predict the radiation status of mice treated with 2 Gy to PH. Similarly, the PB signature of 10 Gy PH failed to predict the radiation status of mice irradiated with 10 Gy to HL. Taken together, these results suggested that the PB molecular response to ionizing radiation is distinct depending upon which portion of the body is exposed. Examination of the genes which comprise the PB signatures of partial body irradiation provide a possible explanation for the lack of predictions across different partial irradiation conditions. We found no overlapping genes between the 3 partial body irradiation conditions at 0.5 or 2 Gy dose levels and only 1 non-annotated gene, RIKEN cDNA 6330579B17, which was in common between the AH, PH and HL groups at 10 Gy dose level. Taken in a broader context, these results demonstrate that partial body irradiation induces unique PB molecular responses as a function of the extent of the exposure and that caution should be applied when applying PB gene signatures to diagnose partially irradiated individuals. Furthermore, the lack of ability to discriminate dose levels across different partial body conditions suggests that distinct reference gene expression profiles may be necessary to accurately predict the radiation status of partially irradiated individuals.

Recently, we and others have sought to develop and validate PB signatures of radiation exposure generated from TBI treatment of mice and/or humans for the purpose of biodosimetry in a mass casualty radiation event [9], [10], [19]. These studies have generated optimism that PB gene expression profiles could be applied in a high throughput fashion as a means of screening or triaging thousands of individuals following a dirty bomb or IND detonation in a large city [9], [10], [37], [38]. Interestingly, independent studies have confirmed the potential accuracy of gene expression profiling to predict radiation status using ex vivo irradiated human PB samples [37] and instruments are currently under development to apply such signatures in the analysis of small blood volumes [39]. Other strategies which have shown promise in biodosimetry include qRT-PCR analysis of specific PB biomarkers (e.g. GADD45)[40], ELISA of multiple blood proteins [41] and urinary metabolomics which utilizes ultra-performance liquid chromatography-time of flight mass spectrometry (UPLC-TOFMS) [42]. However, we show here that PB signatures generated from TBI-treated mice or humans may not be able to predict the radiation status of partially irradiated people or predict the dose level of radiation exposure in such individuals. Similarly, we found very few genes in common between the PB signatures of partial body irradiation and our previously developed PB signatures of TBI (Table S3) and found only 1 gene, *Cdkn1a*, in common with a PB signature of human TBI exposure [19]. The divergent hematologic consequences of partial body irradiation and TBI were also evident in the PB cell content, which revealed substantial depletion of total PB MNCs and B cells in the TBI-treated mice compared to partially irradiated mice. Nevertheless, it is also clear that there is the potential to develop signatures that can accurately predict the partial body radiation events, including the anatomic location of the radiation. While we might have hoped that a simple assay could be used independent of the nature of the radiation exposure, it still could be feasible to employ a collection of signatures as the basis for an assay that assessed the nature and extent of a radiation exposure. In reality, the use of multiple signatures does not make the actual assay more difficult since it is one measure of the full complement of genes that is made and then the activity of the various signatures is measured from this gene expression data. As such, it should be possible to develop an algorithm that evaluated each of the relevant signatures (total body, partial body by site) to then make a determination of the health status of the individual.

In a broader context, the PB radiation signatures that we have developed have the potential to serve as biomarkers of individual susceptibility to radiation-induced toxicity in patients undergoing large volume therapeutic irradiation or TBI. Moreover, sufficient molecular overlap has now been established between normal hematopoietic stem cells and cancer stem cells [43] such that the PB signatures of radiation sensitivity developed here may help to predict the susceptibility of certain cancers to radiation therapy.

## Supporting Information

Table S1 Gene lists for partial body irradiation signatures.
(0.42 MB DOC)

Table S2 Overlapping genes between partial irradiation conditions.
(0.16 MB DOC)

Table S3 Overlapping genes between TBI and Partial Body Signatures.
(0.05 MB DOC)
