# Supplementary material for: Diagnosis of Partial Body Radiation Exposure in Mice Using Peripheral Blood Gene Expression Profiles
Source: PLoS One. 2010 Jul 12;5(7):e11535. doi: 10.1371/journal.pone.0011535 (PMC2902517; doi:10.1371/journal.pone.0011535)
Supplement: Table S1 — Gene lists for partial body irradiation signatures. (0.42 MB DOC) [file pone.0011535.s001.doc]

| **Operon OligoID**  **Table S1. Gene lists for partial body irradiation signatures** | **Gene Symbol** | **RefSeq** | **GenBank** | **Description** |
| --- | --- | --- | --- | --- |
| **Anterior**  **0.5 Gy** |  |  |  |  |
| M400000939 | Mnda | NM_008327 | M31418 | interferon activated gene 202B (Ifi202b) |
| M200003431 | Mmp8 | NM_008611 |  | matrix metalloproteinase 8 |
| M200013923 | Rspondin | NM_138683 | AB016768 | thrombospondin type 1 domain containing gene |
| M200006785 | Pim3 | NM_145478 | BX523241BC017621 | proviral integration site 3 |
| M300001311 | Mrps25 |  | AK131114 |  |
| M200012649 |  | NM_028137 | BC052696 | RIKEN cDNA 5730528L13 gene (5730528L13Rik) |
| M200005961 | Vps29 | NM_019780 |  | vacuolar protein sorting 29 (S. pombe) |
| M400003393 | Rnf149 | XM_129803 |  | PREDICTED: ring finger protein 149 |
| M200007234 | Dnajc12 | NM_013888 | BC071211 | DnaJ (Hsp40) homolog, subfamily C, member 12 |
| M200000016 | D11Ertd99e | NM_026618 | CA450971 | DNA segment, Chr 11, ERATO Doi 99, expressed |
| M300001683 | Q8K0Z1 | XM_126426 | BC075632 | PREDICTED: cDNA sequence BC038313 |
| M200006721 | Mrpl15 | NM_025300 | BC068230 | mitochondrial ribosomal protein L15 |
| M400013899 |  |  |  |  |
| M300007148 | Mrpl51 | NM_025595 |  | mitochondrial ribosomal protein L51 |
| M200009412 | Prg2 | NM_008920 | BY710298 | proteoglycan 2, bone marrow |
| M200013516 |  | NM_021430 | BC051945 | RIKEN cDNA 2900002H16 gene (2900002H16Rik) |
| M400006409 |  |  | CN694825 |  |
| M200001873 | Irg1 | XM_127883 | L38281 | PREDICTED: immunoresponsive gene 1 |
| M200003371 | Tnfrsf11b | NM_008764 | AB013898 | tumor necrosis factor receptor superfamily, member 11b (osteoprotegerin) |
| M300022296 |  | NM_172623 |  | triggering receptor expressed on myeloid cells-like 4 |
| M200001752 | Ahr | NM_013464 |  | aryl-hydrocarbon receptor |
| M200003102 | Hs3st1 | NM_010474 | AF019385 | heparan sulfate (glucosamine) 3-O-sulfotransferase 1 |
| M400001468 |  | XM_135176 |  | PREDICTED: RIKEN cDNA 4921524K10 gene |
| M400001965 | Nxt1 | NM_019761 | AA915380 | NTF2-related export protein 1 |
| M200000053 | Fcgr1 | NM_010186 |  | Fc receptor, IgG, high affinity I |
| M300004285 | Slc15a3 | NM_023044 | AF121080 | solute carrier family 15, member 3 |
| M200006408 | Lmo2 | NM_008505 | BC057880 | LIM domain only 2 |
| M200002206 | Prss19 | NM_008940 | D30785 | protease, serine, 19 (neuropsin) |
| M300000427 | Ixl | NM_026042 | BC023192 | intersex-like (Drosophila) |
| M300021034 | Lgals3 | NM_010705 | BI078701 | lectin, galactose binding, soluble 3 |
| M400004051 | Zfp622 | NM_144523 |  | zinc finger protein 622 |
| M300002923 | Emb | NM_010330 |  | embigin |
| M200014304 | Q9D2U5 |  |  |  |
| M400005620 | Nfil3 | NM_017373 |  | nuclear factor, interleukin 3, regulated |
| M200007726 | Siglec5 | NM_145581 |  | sialic acid binding Ig-like lectin 5 |
| M400002756 |  |  |  |  |
| M300021033 | Lgals3 | NM_010705 |  | lectin, galactose binding, soluble 3 |
| M300003720 | Clecsf10 | NM_020001 | BC023008 | C-type (calcium dependent, carbohydrate recognition domain) lectin, superfamily member 10 |
| M200001695 | Tnfaip2 | NM_009396 |  | tumor necrosis factor, alpha-induced protein 2 |
| M200013611 |  | NM_025915 |  | RIKEN cDNA 2600017H02 gene (2600017H02Rik) |
| M400002204 | IRS2_MOUSE | XM_357863 |  | PREDICTED: similar to Insulin receptor substrate-2 (IRS-2) (4PS) (LOC384783) |
| M400000940 | Ifi16 | NM_008329 | M31419 | interferon, gamma-inducible protein 16 |
| M300006107 | Tpm2 | NM_009416 | M81086 | tropomyosin 2, beta |
| M200003538 | Smpdl3b | NM_133888 | BC009087 | sphingomyelin phosphodiesterase, acid-like 3B |
| M200005413 | Ndufa11 | XM_128696 | BC053075 | PREDICTED: NADH dehydrogenase (ubiquinone) 1 alpha subcomplex |
| M300005418 | Il1rn | NM_031167 | BC042532 | interleukin 1 receptor antagonist |
| M400008538 |  |  |  |  |
| M400000265 |  |  |  |  |
| M400014572 |  |  |  |  |
| M400000722 | Q91VU5 |  | AK129167 |  |
|  |  |  |  |  |
| **Anterior**  **2.0 Gy** |  |  |  |  |
| M400000939 | Mnda | NM_008327 | M31418 | interferon activated gene 202B (Ifi202b) |
| M200013923 | Rspondin | NM_138683 | AB016768 | thrombospondin type 1 domain containing gene |
| M200013516 |  | NM_021430 | BC051945 | RIKEN cDNA 2900002H16 gene (2900002H16Rik) |
| M200012649 |  | NM_028137 | BC052696 | RIKEN cDNA 5730528L13 gene (5730528L13Rik) |
| M400002204 | IRS2_MOUSE | XM_357863 |  | PREDICTED: similar to Insulin receptor substrate-2 (IRS-2) (4PS) (LOC384783) |
| M200003102 | Hs3st1 | NM_010474 | AF019385 | heparan sulfate (glucosamine) 3-O-sulfotransferase 1 |
| M400013899 |  |  |  |  |
| M200001752 | Ahr | NM_013464 |  | aryl-hydrocarbon receptor |
| M200006721 | Mrpl15 | NM_025300 | BC068230 | mitochondrial ribosomal protein L15 |
| M400001965 | Nxt1 | NM_019761 | AA915380 | NTF2-related export protein 1 |
| M200006785 | Pim3 | NM_145478 | BX523241  BC017621 | proviral integration site 3 |
| M200007726 | Siglec5 | NM_145581 |  | sialic acid binding Ig-like lectin 5 |
| M300004285 | Slc15a3 | NM_023044 | AF121080 | solute carrier family 15, member 3 |
| M300002923 | Emb | NM_010330 |  | embigin |
| M200002206 | Prss19 | NM_008940 | D30785 | protease, serine, 19 (neuropsin) |
| M300005418 | Il1rn | NM_031167 | BC042532 | interleukin 1 receptor antagonist |
| M200005961 | Vps29 | NM_019780 |  | vacuolar protein sorting 29 (S. pombe) |
| M200013611 |  | NM_025915 |  | RIKEN cDNA 2600017H02 gene (2600017H02Rik) |
| M300021034 | Lgals3 | NM_010705 | BI078701 | lectin, galactose binding, soluble 3 |
| M400005620 | Nfil3 | NM_017373 |  | nuclear factor, interleukin 3, regulated |
| M400002756 |  |  |  |  |
| M300003720 | Clecsf10 | NM_020001 | BC023008 | C-type (calcium dependent, carbohydrate recognition domain) lectin, superfamily member 10 |
| M200003538 | Smpdl3b | NM_133888 | BC009087 | sphingomyelin phosphodiesterase, acid-like 3B |
| M300021033 | Lgals3 | NM_010705 |  | lectin, galactose binding, soluble 3 |
| M400000940 | Ifi16 | NM_008329 | M31419 | interferon, gamma-inducible protein 16 |
|  |  |  |  |  |
| **Anterior 10.0 Gy** |  |  |  |  |
| M200009688 | Sertad1 | NM_018820 | AF366401 | SERTA domain containing 1 (Sertad1) |
| M200002206 | Prss19 | NM_008940 | D30785 | protease, serine, 19 (neuropsin) |
| M400015356 |  |  |  |  |
| M200014304 | Q9D2U5 |  |  |  |
| M200000524 | Il1r2 | NM_010555 | X59769 | interleukin 1 receptor, type II |
| M200006000 | Hat1 | NM_026115 |  | histone aminotransferase 1 |
| M400005620 | Nfil3 | NM_017373 |  | nuclear factor, interleukin 3, regulated |
| M200007678 | Dusp10 | NM_022019 |  | dual specificity phosphatase 10 (Dusp10) |
| M400001091 | Pde4b | NM_019840 | AF208023 | phosphodiesterase 4B, cAMP specific |
| M300006203 | PPCS_MOUSE | NM_026494 |  | RIKEN cDNA 6330579B17 gene (6330579B17Rik) |
| M400004051 | Zfp622 | NM_144523 |  | zinc finger protein 622 |
| M200002903 | Fusip1 | NM_010178 | AF042383 | FUS interacting protein (serine-arginine rich) 1 |
| M400003393 | Rnf149 | XM_129803 |  | PREDICTED: ring finger protein 149 |
| M300000599 | Ly9 |  | BC055380 |  |
| M400013710 |  |  | BM937289 |  |
| M200007439 | Igtp | NM_019440 | AK128991 | interferon inducible GTPase 2 (Iigp2) |
| M400008831 | CMF9_MOUSE |  |  |  |
| M200015749 | Maff | NM_010755 | BC022952 | v-maf musculoaponeurotic fibrosarcoma oncogene family, protein F (avian) |
| M300001678 |  | NM_033562 |  | Der1-like domain family, member 2 |
| M300002284 |  | NM_134052 | BC005695 | expressed sequence AL024210 (AL024210) |
| M200000053 | Fcgr1 | NM_010186 |  | Fc receptor, IgG, high affinity I |
| M300002112 |  | NM_172391 |  | RIKEN cDNA 1110064P04 gene (1110064P04Rik) |
| M200002772 | Pole2 | NM_011133 | BC063772 | polymerase (DNA directed), epsilon 2 (p59 subunit) |
| M300006854 | Sec8l1 |  |  |  |
| M200004109 | Actr6 | NM_025914 | BC062137 | ARP6 actin-related protein 6 homolog (yeast) |
| M200009441 | MEFV_MOUSE | NM_019453 | AF143409 | Mediterranean fever (Mefv) |
| M200013484 | CMF9_MOUSE | NM_145448 |  | RIKEN cDNA 9030617O03 gene (9030617O03Rik) |
| M400008510 | Igl-V1 |  |  |  |
| M400000048 | Bbc3 | NM_133234 | AF332560 | Bcl-2 binding component 3 (Bbc3) |
| M200005878 | Crot | NM_023733 | BC012308 | carnitine O-octanoyltransferase (Crot) |
| M200001752 | Ahr | NM_013464 |  | aryl-hydrocarbon receptor (Ahr) |
| M400011350 |  | NM_019976 | BF723196BC008994 | RIKEN cDNA 5430413I02 gene (5430413I02Rik) |
| M400006069 | H2-T23 |  |  |  |
| M200012683 | Acat2 | NM_009338 | CB056472 | acetyl-Coenzyme A acetyltransferase 2 |
| M200013923 | Rspondin | NM_138683 | AB016768 | thrombospondin type 1 domain containing gene |
| M300004550 | Apex2 | NM_029943 |  | apurinic/apyrimidinic endonuclease 2 |
| M200013095 | Pgam2 | NM_018870 |  | phosphoglycerate mutase 2 |
| M200004676 | Cyp2s1 | NM_028775 |  | cytochrome P450, family 2, subfamily s, polypeptide 1 |
| M200003431 | Mmp8 | NM_008611 |  | matrix metalloproteinase 8 |
| M400003169 |  | NM_177887 |  | cDNA sequence BC022651 (BC022651) |
| M200004083 | Shprh |  |  |  |
| M400011010 | Fes | NM_010194 |  | feline sarcoma oncogene |
| M200013634 |  | NM_144905 |  | RIKEN cDNA 6330416G13 gene (6330416G13Rik) |
| M200004718 | Popdc2 | NM_022318 | BC064005 | popeye domain containing 2 |
| M400001965 | Nxt1 | NM_019761 | AA915380 | NTF2-related export protein 1 |
| M400013713 |  |  |  |  |
| M200006087 | Cdca4 | NM_028023 | BC055824 | cell division cycle associated 4 |
| M200014180 | Dhrs6 | NM_027208 | BC021461 | dehydrogenase/reductase (SDR family) member 6 |
| M300021033 | Lgals3 | NM_010705 |  | lectin, galactose binding, soluble 3 |
| M400008488 |  |  |  |  |

| **Operon OligoID** | **Gene Symbol** | **RefSeq** | **GenBank** | **Description** |
| --- | --- | --- | --- | --- |
| **Posterior**  **0.5 Gy** |  |  |  |  |
| M300004428 | Ccnj | NM_172839 |  | cyclin J |
| M200000053 | Fcgr1 | NM_010186 |  | Fc receptor, IgG, high affinity I |
| M400000939 | Mnda | NM_008327 | M31418 | interferon activated gene 202B (Ifi202b) |
| M200003455 | Lamp1 | NM_010684 | BC049097 | lysosomal membrane glycoprotein 1 |
| M400008661 | Ugt1a1 | NM_201642 |  | UDP glycosyltransferase 1 family, polypeptide A10 |
| M400000940 | Ifi16 | NM_008329 | M31419 | interferon, gamma-inducible protein 16 |
| M400001228 | Clecsf12 | NM_020008 | AF262985 | C-type (calcium dependent, carbohydrate recognition domain) lectin, superfamily member 12 |
| M200004905 | Dock9 |  | AK122431 |  |
| M200007149 |  | NM_146155 | BC060231 | RIKEN cDNA D030015G18 gene (D030015G18Rik) |
| M300006738 | Oasl2 | NM_011854 | AF068835 | 2'-5' oligoadenylate synthetase-like 2 |
| M200000522 | Hlx | NM_008250 | X58250 | H2.0-like homeo box 1 (Drosophila) |
| M300021989 | Olfr645 | NM_207144 |  | olfactory receptor 645 |
| M200012232 | Rad9 | NM_011237 | AF045663 | RAD9 homolog (S. pombe) |
| M300007079 | Ptpn15 |  | AF135166 |  |
| M200007653 | Pdha1 | NM_008810 | BC007142 | pyruvate dehydrogenase E1 alpha 1 |
| M200006276 | Fbxw4 | NM_013907 | AB022163 | F-box and WD-40 domain protein 4 |
| M200001333 |  | NM_007421 | BC039943 | adenylosuccinate synthetase like 1 (Adssl1) |
| M300005669 | Tpd52 | NM_009412 |  | tumor protein D52 |
| M300021033 | Lgals3 | NM_010705 |  | lectin, galactose binding, soluble 3 |
| M400003169 |  | NM_177887 |  | cDNA sequence BC022651 (BC022651) |
| M200015234 | Cstf2 | NM_133196 |  | cleavage stimulation factor, 3' pre-RNA subunit 2 |
| M200003624 | H3f3b | NM_008211 | BI151114 | H3 histone, family 3B |
| M200013596 | Arrdc4 | NM_025549 |  | arrestin domain containing 4 |
| M400002605 |  | NM_175465 |  | SEC14 and spectrin domains 1 (Sestd1) |
| M300007417 | APHC_MOUSE | NM_025408 | BC023924 | phytoceramidase, alkaline (Phca)" |
|  |  |  |  |  |
| **Posterior 2.0 Gy** |  |  |  |  |
| M300009937 | Galm | NM_176963 |  | galactose mutarotase (Galm) |
| M400001228 | Clecsf12 | NM_020008 | AF262985 | C-type (calcium dependent, carbohydrate recognition domain) lectin, superfamily member 12 |
| M200008541 | Jundm2 | NM_030887 | AB077438 | Jun dimerization protein 2 (Jundm2) |
| M200005601 |  |  |  |  |
| M200008143 | Zfp7 | NM_145916 | BC062103 | zinc finger protein 7 (Zfp7) |
| M200007653 | Pdha1 | NM_008810 | BC007142 | pyruvate dehydrogenase E1 alpha 1 (Pdha1) |
| M200015166 | Fgd4 | NM_139232 |  | FYVE, RhoGEF and PH domain containing 4 |
| M200000053 | Fcgr1 | NM_010186 |  | Fc receptor, IgG, high affinity I |
| M200004154 |  | NM_028658 |  | RIKEN cDNA 1110018J12 gene (1110018J12Rik) |
| M400002781 | Gp49a |  |  |  |
| M300002335 | Arf2 | NM_007477 |  | ADP-ribosylation factor 2 |
| M200013566 | Gng12 | NM_025278 |  | guanine nucleotide binding protein (G protein), gamma 12 |
| M200007149 |  | NM_146155 | BC060231 | RIKEN cDNA D030015G18 gene (D030015G18Rik) |
| M200011988 | Q8CIB2 | XM_355643 |  | PREDICTED: RIKEN cDNA 0610007L01 gene (0610007L01Rik) |
| M300017143 | Plaur | NM_011113 | BC010309 | urokinase plasminogen activator receptor |
| M200000522 | Hlx | NM_008250 | X58250 | H2.0-like homeo box 1 (Drosophila) (Hlx1) |
| M200004109 | Actr6 | NM_025914 | BC062137 | ARP6 actin-related protein 6 homolog (yeast) |
| M200005430 | Mospd2 |  |  |  |
| M400005620 | Nfil3 | NM_017373 |  | nuclear factor, interleukin 3, regulated |
| M400002615 | Snag1 | NM_130796 | BC063089 | sorting nexin associated golgi protein 1 |
| M300021033 | Lgals3 | NM_010705 |  | lectin, galactose binding, soluble 3 |
| M200002437 |  | NM_026189 |  | RIKEN cDNA 2310005P05 gene (2310005P05Rik) |
| M200002445 | Surf1 | NM_013677 |  | surfeit gene 1 |
| M200001367 | Nr4a2 | NM_013613 |  | nuclear receptor subfamily 4, group A, member 2 |
| M200002536 | Per1 | NM_011065 | AF022992 | period homolog 1 (Drosophila) |
|  |  |  |  |  |
| **Posterior 10.0 Gy** |  |  |  |  |
| M300006203 | PPCS_MOUSE | NM_026494 |  | RIKEN cDNA 6330579B17 gene (6330579B17Rik) |
| M200013064 | Rasa2 | NM_053268 |  | RAS p21 protein activator 2 |
| M300006227 | Plk3 | NM_013807 | BC031180 | polo-like kinase 3 (Drosophila) |
| M200014304 | Q9D2U5 |  |  |  |
| M400011188 | Prdx5 | NM_012021 |  | peroxiredoxin 5 |
| M200004109 | Actr6 | NM_025914 | BC062137 | ARP6 actin-related protein 6 homolog (yeast) |
| M200003109 | Nol8 | XM_484255 | BC058699 | PREDICTED: nucleolar protein 8 |
| M200006364 | Dcxr | NM_026428 |  | dicarbonyl L-xylulose reductase |
| M300005377 | Brd3 | NM_023336 | BC031536 | bromodomain containing 3 |
| M400010983 | Ccng1 | NM_009831 | AK128957 | cyclin G1 |
| M200005601 |  |  |  |  |
| M300012623 | Bcor |  | BC058656 |  |
| M300002112 |  | NM_172391 |  | RIKEN cDNA 1110064P04 gene (1110064P04Rik) |
| M200004941 |  | NM_023397 |  | RIKEN cDNA 1810034K20 gene (1810034K20Rik) |
| M200000800 | Ccng1 |  | AK128957 |  |
| M200013484 | CMF9_MOUSE | NM_145448 |  | RIKEN cDNA 9030617O03 gene (9030617O03Rik) |
| M200012232 | Rad9 | NM_011237 | AF045663 | RAD9 homolog (S. pombe) |
| M200008541 | Jundm2 | NM_030887 | AB077438 | Jun dimerization protein 2 |
| M200015749 | Maff | NM_010755 | BC022952 | v -maf musculoaponeurotic fibrosarcoma oncogene family, protein F (avian) |
| M400005620 | Nfil3 | NM_017373 |  | nuclear factor, interleukin 3, regulated |
| M300005614 |  | NM_027093 |  | RIKEN cDNA 2310003L22 gene (2310003L22Rik) |
| M400001965 | Nxt1 | NM_019761 | AA915380 | NTF2-related export protein 1 |
| M200007578 | Cdkn1a | NM_007669 |  | cyclin-dependent kinase inhibitor 1A (P21) |
| M200006246 | Rhou | NM_133955 | AB051827 | ras homolog gene family, member U |
| M400006398 | Serhl | NM_023475 | AJ245737 | serine hydrolase-like |

| **Operon OligoID** | **Gene Symbol** | **RefSeq** | **GenBank** | **Description** |
| --- | --- | --- | --- | --- |
| **Hind Limb**  **0.5 Gy** |  |  |  |  |
| M300007590 | C81234 | NM_172479 |  | solute carrier family 38, member 5 (Slc38a5) |
| M200002723 | Cbx4 | NM_007625 | U63387 | chromobox homolog 4 (Drosophila Pc class) |
| M200004995 | Il1rap | NM_008364 |  | interleukin 1 receptor accessory protein (Il1rap), transcript variant 1 |
| M300015973 | Olfr669 | NM_147043 | CB173475 | olfactory receptor 669 |
| M400005909 | Olfr804 |  |  |  |
| M300019564 |  | NM_173408 | BC030335 | cDNA sequence BC030335 (BC030335) |
| M200004576 | Q9DAY1 | XM_484930 |  | PREDICTED: RIKEN cDNA 1600012P17 gene (1600012P17Rik) |
| M300016643 | Q8CF37 | XM_485737 |  | PREDICTED: RIKEN cDNA 1700023L04 gene (1700023L04Rik) |
| M400014572 |  |  |  |  |
| M200002478 | Nudt14 | NM_025399 |  | nudix (nucleoside diphosphate linked moiety X)-type motif 14 |
| M400002539 |  |  |  |  |
| M400002770 | Q923B5 |  | BC006653 |  |
| M200003725 | Pscd3 | NM_011182 | BC035296 | pleckstrin homology, Sec7 and coiled-coil domains 3 |
| M200007100 | N155_MOUSE | NM_133227 | AK129217 | nucleoporin 155 (Nup155) |
| M300009299 |  | NM_026454 | BC016117 | RIKEN cDNA 2510010F15 gene (2510010F15Rik) |
| M300012133 |  | NM_175132 |  | synaptopodin 2-like (Synpo2l) |
| M200014069 | Baalc |  |  |  |
| M400001325 |  | NM_146197 |  | cDNA sequence BC031140 (BC031140) |
| M400006052 |  | XM_356705 |  | PREDICTED: similar to 60S ribosomal protein L19 (LOC382844) |
| M200009524 | OPT_MOUSE | NM_054076 | AF333980 | opticin (Optc) |
| M400012797 | Es2el |  | BC013711 |  |
| M400002581 |  | NM_026394 |  | RIKEN cDNA 1110055J05 gene (1110055J05Rik) |
| M400001818 | Wdr20 | NM_027614 |  | WD repeat domain 20 |
| M300020418 | Olfr1234 | NM_146455 |  | olfactory receptor 1241 |
| M300006363 | Ptpru | NM_011214 | U55057 | protein tyrosine phosphatase, receptor type, U |
| M300019567 |  | NM_177020 |  | RIKEN cDNA E030011O05 gene (E030011O05Rik) |
| M200001988 | Sgne1 | NM_009162 |  | secretory granule neuroendocrine protein 1, 7B2 protein |
| M300012538 | Maob | NM_172778 |  | monoamine oxidase B |
| M300015472 |  |  | AB095745 |  |
| M200005359 | Gjb5 | NM_010291 |  | gap junction membrane channel protein beta 5 |
| M300013305 | Lhfp | NM_178358 | BC051434 | lipoma HMGIC fusion partner-like 1 (Lhfpl1) |
| M200007299 | Slc41a3 | XM_132686 |  | PREDICTED: solute carrier family 41, member 3 |
| M200015412 | CLDM_MOUSE |  |  |  |
| M200016183 | CLDG_MOUSE | NM_053241 |  | claudin 16 (Cldn16) |
| M200015419 | Hsf2bp | XM_484622 |  | PREDICTED: heat shock transcription factor 2 binding protein |
| M300000459 |  | NM_177214 | BC063261 | activating signal cointegrator 1 complex subunit 3-like 1 (Ascc3l1) |
| M300014222 |  | NM_177761 |  | cDNA sequence BC022713 (BC022713) |
| M400019380 |  |  |  |  |
| M300008277 | Ptgs2 | NM_011198 | M88242 | prostaglandin-endoperoxide synthase 2 |
| M200003431 | Mmp8 | NM_008611 |  | matrix metalloproteinase 8 |
| M300009352 |  | NM_178715 |  | transmembrane protein 30B (Tmem30b) |
| M400004295 | Trim10 | NM_011280 | AF220121 | tripartite motif protein 10 |
| M200004905 | Dock9 |  | AK122431 |  |
| M300014790 |  | NM_177653 |  | RIKEN cDNA F830045P16 gene (F830045P16Rik) |
| M300005501 | Cat |  |  |  |
| M300019715 | Olfr284 | NM_146281 |  | olfactory receptor 284 |
| M200009409 | Cuzd1 | NM_008411 | U69699 | CUB and zona pellucida-like domains 1 |
| M200015354 | Catsper2 | NM_153075 | BC034181 | cation channel, sperm associated 2 |
| M200003685 |  | NM_027918 |  | RIKEN cDNA 1300017J02 gene (1300017J02Rik) |
| M300021827 | Q8CBC9 |  |  |  |
|  |  |  |  |  |
| **Hind Limb 2.0 Gy** |  |  |  |  |
| M200007299 | Slc41a3 | XM_132686 |  | PREDICTED: solute carrier family 41, member 3 |
| M300021075 | Il31ra | NM_139299 | AB083111 | interleukin 31 receptor A |
| M200006529 | Capn10 | NM_011796 | AF089089 | calpain 10 |
| M400005752 |  | NM_213729 |  | expressed sequence AI842396 (AI842396) |
| M300004812 | Centa1 | NM_172723 |  | centaurin, alpha 1 |
| M200015061 | Q9D2I7 |  |  |  |
| M200012232 | Rad9 | NM_011237 | AF045663 | RAD9 homolog (S. pombe) |
| M200007724 |  | NM_028721 | AY259499 | nephronophthisis 3 (adolescent) (Nphp3) |
| M200001256 | Gas7 | NM_008088 |  | growth arrest specific 7 |
| M400017940 |  |  |  |  |
| M200003339 | Sdcbp | NM_016807 |  | syndecan binding protein |
| M400005175 | Maf |  |  |  |
| M200014188 | Hemk1 | NM_133984 |  | HemK methyltransferase family member 1 |
| M200005915 | Ncoa4 | NM_019744 | AK129020 | nuclear receptor coactivator 4 |
| M400014572 |  |  |  |  |
| M200003725 | Pscd3 | NM_011182 | BC035296 | pleckstrin homology, Sec7 and coiled-coil domains 3 |
| M400004051 | Zfp622 | NM_144523 |  | zinc finger protein 622 |
| M400011251 | Zfp95 | NM_016683 | AK173086 | zinc finger protein 95 |
| M400012207 | Cpne1 | NM_170588  NM_170590 |  | copine I (Cpne1), transcript variant 1 |
| M200014557 |  | XM_127336 |  | PREDICTED: SECIS binding protein 2 (Secisbp2) |
| M200001988 | Sgne1 | NM_009162 |  | secretory granule neuroendocrine protein 1, 7B2 protein |
| M200007813 | Q8R571 | NM_001004156 | AK129198 | cDNA sequence BC023181 (BC023181) |
| M200007727 | Fbf1 | NM_172571 | BC043126 | Fas (TNFRSF6) binding factor 1 |
| M200004076 | Q8BR47 | XM_484702 | BC076613 | PREDICTED: RIKEN cDNA A930014I12 gene (A930014I12Rik) |
| M200009937 |  | NM_025670 |  | RIKEN cDNA 5730403B10 gene (5730403B10Rik) |
|  |  |  |  |  |
| **Hind Limb**  **10.0 Gy** |  |  |  |  |
| M400005411 |  | NM_145916 | BC062103 | zinc finger protein 7 (Zfp7) |
| M300001057 | Apobec3 | NM_030255 |  | apolipoprotein B editing complex 3 |
| M300021075 | Il31ra | NM_139299 | AB083111 | interleukin 31 receptor A |
| M200000052 | Rad52 | NM_011236 | Z32767 | RAD52 homolog (S. cerevisiae) |
| M200007299 | Slc41a3 | XM_132686 |  | PREDICTED: solute carrier family 41, member 3 |
| M200004830 | Arrdc2 | XM_134227 |  | PREDICTED: arrestin domain containing 2 |
| M200003339 | Sdcbp | NM_016807 |  | syndecan binding protein |
| M300003442 | Cd200 |  | BC054759 |  |
| M200013566 | Gng12 | NM_025278 |  | guanine nucleotide binding protein (G protein), gamma 12 |
| M400002516 | Serpina12 | NM_026535 |  | serine (or cysteine) proteinase inhibitor, clade A (alpha-1 antiproteinase, antitrypsin), member 12 |
| M200008116 |  | NM_028766 |  | RIKEN cDNA 1200015A22 gene (1200015A22Rik) |
| M200008143 | Zfp7 | NM_145916 | BC062103 | zinc finger protein 7 |
| M200007112 | Zfp87 | NM_133228 | BC031988 | zinc finger protein 87 (Zfp87) |
| M400006153 | D5Ertd606e |  | BC064466 |  |
| M200003725 | Pscd3 | NM_011182 | BC035296 | pleckstrin homology, Sec7 and coiled-coil domains 3 |
| M300006203 | PPCS_MOUSE | NM_026494 |  | RIKEN cDNA 6330579B17 gene (6330579B17Rik) |
| M200014557 |  | XM_127336 |  | PREDICTED: SECIS binding protein 2 (Secisbp2) |
| M200012232 | Rad9 | NM_011237 | AF045663 | RAD9 homolog (S. pombe) |
| M200009550 | Ppp1r3f | NM_138605 | BC059275 | protein phosphatase 1, regulatory (inhibitor) subunit 3F |
| M200003538 | Smpdl3b | NM_133888 | BC009087 | sphingomyelin phosphodiesterase, acid-like 3B |
| M400000025 | Ccl3 | NM_011337 | AA895994 | chemokine (C-C motif) ligand 3 |
| M400014572 |  |  |  |  |
| M200004676 | Cyp2s1 | NM_028775 |  | cytochrome P450, family 2, subfamily s, polypeptide 1 |
| M300001073 | Rps25 |  |  |  |
| M200009937 |  | NM_025670 |  | RIKEN cDNA 5730403B10 gene (5730403B10Rik) |
