# Supplementary material for: Diagnosis of Partial Body Radiation Exposure in Mice Using Peripheral Blood Gene Expression Profiles
Source: PLoS One. 2010 Jul 12;5(7):e11535. doi: 10.1371/journal.pone.0011535 (PMC2902517; doi:10.1371/journal.pone.0011535)
Supplement: Table S2 — Overlapping genes between partial irradiation conditions. (0.16 MB DOC) [file pone.0011535.s002.doc]

**Table S2**. Overlapping genes between partial irradiation conditions

|  | **Gene Symbol** | **RefSeq** | **GenBank** | **Description** |
| --- | --- | --- | --- | --- |
| **Anterior and Posterior 0.5 Gy** |  |  |  |  |
| M200000053 | Fcgr1 | NM_010186 |  | Fc receptor, IgG, high affinity I |
| M300021033 | Lgals3 | NM_010705 |  | lectin, galactose binding, soluble 3 |
| M400000939 | Mnda | NM_008327 | M31418 | interferon activated gene 202B (Ifi202b) |
| M400000940 | Ifi16 | NM_008329 | M31419 | interferon, gamma-inducible protein 16 |
| **Anterior and Posterior 2.0 Gy** |  |  |  |  |
| M300021033 | Lgals3 | NM_010705 |  | lectin, galactose binding, soluble 3 |
| M400005620 | Nfil3 | NM_017373 |  | nuclear factor, interleukin 3, regulated |
| **Anterior and Posterior 10.0 Gy** |  |  |  |  |
| M200004109 | Actr6 | NM_025914 | BC062137 | ARP6 actin-related protein 6 homolog (yeast) |
| M200013484 | CMF9_MOUSE | NM_145448 |  | RIKEN cDNA 9030617O03 gene (9030617O03Rik) |
| M200014304 | Q9D2U5 |  |  |  |
| M200015749 | Maff | NM_010755 | BC022952 | v-maf musculoaponeurotic fibrosarcoma oncogene family, protein F (avian) |
| M300002112 |  | NM_172391 |  | RIKEN cDNA 1110064P04 gene (1110064P04Rik) |
| M300006203 | PPCS_MOUSE | NM_026494 |  | RIKEN cDNA 6330579B17 gene (6330579B17Rik) |
| M400001965 | Nxt1 | NM_019761 | AA915380 | NTF2-related export protein 1 |
| M400005620 | Nfil3 | NM_017373 |  | nuclear factor, interleukin 3, regulated |
| **Anterior and Hind Limb 0.5 Gy** |  |  |  |  |
| M200003431 | Mmp8 | NM_008611 |  | matrix metalloproteinase 8 |
| M400014572 |  |  |  |  |
| **Anterior and Hind Limb 2.0 Gy** |  |  |  |  |
| NONE |  |  |  |  |
| **Anterior and Hind Limb 10.0 Gy** |  |  |  |  |
| M200004676 | Cyp2s1 | NM_028775 |  | cytochrome P450, family 2, subfamily s, polypeptide 1 |
| M300006203 | PPCS_MOUSE | NM_026494 |  | RIKEN cDNA 6330579B17 gene (6330579B17Rik) |
| **Posterior and Hind Limb 0.5 Gy** |  |  |  |  |
| M200004905 | Dock9 |  | AK122431 |  |
| **Posterior and Hind Limb 2.0 Gy** |  |  |  |  |
| NONE |  |  |  |  |
| **Posterior and Hind Limb 10.0 Gy** |  |  |  |  |
| M200012232 | Rad9 | NM_011237 | AF045663 | RAD9 homolog (S. pombe) |
| M300006203 | PPCS_MOUSE | NM_026494 |  | RIKEN cDNA 6330579B17 gene (6330579B17Rik) |

| **Operon OligoID** | **Gene Symbol** | **RefSeq** | **GenBank** | **Description** |
| --- | --- | --- | --- | --- |
| **Anterior 0.5Gy and 2.0 Gy** |  |  |  |  |
| M200001752 | Ahr | NM_013464 |  | aryl-hydrocarbon receptor |
| M200002206 | Prss19 | NM_008940 | D30785 | protease, serine, 19 (neuropsin) |
| M200003102 | Hs3st1 | NM_010474 | AF019385 | heparan sulfate (glucosamine) 3-O-sulfotransferase 1 |
| M200003538 | Smpdl3b | NM_133888 | BC009087 | sphingomyelin phosphodiesterase, acid-like 3B |
| M200005961 | Vps29 | NM_019780 |  | vacuolar protein sorting 29 (S. pombe) |
| M200006721 | Mrpl15 | NM_025300 | BC068230 | mitochondrial ribosomal protein L15 |
| M200006785 | Pim3 | NM_145478 | BX523241,BC017621 | proviral integration site 3 |
| M200007726 | Siglec5 | NM_145581 |  | sialic acid binding Ig-like lectin 5 |
| M200012649 |  | NM_028137 | BC052696 | RIKEN cDNA 5730528L13 gene (5730528L13Rik) |
| M200013516 |  | NM_021430 | BC051945 | RIKEN cDNA 2900002H16 gene (2900002H16Rik) |
| M200013611 |  | NM_025915 |  | RIKEN cDNA 2600017H02 gene (2600017H02Rik) |
| M200013923 | Rspondin | NM_138683 | AB016768 | thrombospondin type 1 domain containing gene |
| M300002923 | Emb | NM_010330 |  | embigin |
| M300003720 | Clecsf10 | NM_020001 | BC023008 | C-type (calcium dependent, carbohydrate recognition domain) lectin, superfamily member 10 |
| M300004285 | Slc15a3 | NM_023044 | AF121080 | solute carrier family 15, member 3 |
| M300005418 | Il1rn | NM_031167 | BC042532 | interleukin 1 receptor antagonist |
| M300021033 | Lgals3 | NM_010705 |  | lectin, galactose binding, soluble 3 |
| M300021034 | Lgals3 | NM_010705 | BI078701 | lectin, galactose binding, soluble 3 |
| M400000939 | Mnda | NM_008327 | M31418 | interferon activated gene 202B (Ifi202b) |
| M400000940 | Ifi16 | NM_008329 | M31419 | interferon, gamma-inducible protein 16 |
| M400001965 | Nxt1 | NM_019761 | AA915380 | NTF2-related export protein 1 (Nxt1) |
| M400002204 | IRS2_MOUSE | XM_357863 |  | PREDICTED: similar to Insulin receptor substrate-2 (IRS-2) (4PS) (LOC384783) |
| M400002756 |  |  |  |  |
| M400005620 | Nfil3 | NM_017373 |  | nuclear factor, interleukin 3, regulated (Nfil3) |
| M400013899 |  |  |  |  |
| **Anterior 2.0 Gy and 10.0 Gy** |  |  |  |  |
| M200001752 | Ahr | NM_013464 |  | aryl-hydrocarbon receptor |
| M200002206 | Prss19 | NM_008940 | D30785 | protease, serine, 19 (neuropsin) |
| M200013923 | Rspondin | NM_138683 | AB016768 | thrombospondin type 1 domain containing gene |
| M300021033 | Lgals3 | NM_010705 |  | lectin, galactose binding, soluble 3 |
| M400001965 | Nxt1 | NM_019761 | AA915380 | NTF2-related export protein 1 |
| M400005620 | Nfil3 | NM_017373 |  | nuclear factor, interleukin 3, regulated |
| **Anterior 0.5 Gy and 10.0 Gy** |  |  |  |  |
| M200000053 | Fcgr1 | NM_010186 |  | Fc receptor, IgG, high affinity I |
| M200001752 | Ahr | NM_013464 |  | aryl-hydrocarbon receptor (Ahr) |
| M200002206 | Prss19 | NM_008940 | D30785 | protease, serine, 19 (neuropsin) |
| M200003431 | Mmp8 | NM_008611 |  | matrix metalloproteinase 8 |
| M200013923 | Rspondin | NM_138683 | AB016768 | thrombospondin type 1 domain containing gene |
| M200014304 | Q9D2U5 |  |  |  |
| M300021033 | Lgals3 | NM_010705 |  | lectin, galactose binding, soluble 3 |
| M400001965 | Nxt1 | NM_019761 | AA915380 | NTF2-related export protein 1 |
| M400003393 | Rnf149 | XM_129803 |  | PREDICTED: ring finger protein 149 |
| M400004051 | Zfp622 | NM_144523 |  | zinc finger protein 622 |
| M400005620 | Nfil3 | NM_017373 |  | nuclear factor, interleukin 3, regulated |
| **Posterior 0.5 Gy and 2.0 Gy** |  |  |  |  |
| M200000053 | Fcgr1 | NM_010186 |  | Fc receptor, IgG, high affinity I |
| M200000522 | Hlx | NM_008250 | X58250 | H2.0-like homeo box 1 (Drosophila) (Hlx1) |
| M200007149 |  | NM_146155 | BC060231 | RIKEN cDNA D030015G18 gene (D030015G18Rik) |
| M200007653 | Pdha1 | NM_008810 | BC007142 | pyruvate dehydrogenase E1 alpha 1 |
| M300021033 | Lgals3 | NM_010705 |  | lectin, galactose binding, soluble 3 |
| M400001228 | Clecsf12 | NM_020008 | AF262985 | C-type (calcium dependent, carbohydrate recognition domain) lectin, superfamily member 12 |
| **Posterior 2.0 Gy and 10.0 Gy** |  |  |  |  |
| M200004109 | Actr6 | NM_025914 | BC062137 | ARP6 actin-related protein 6 homolog (yeast) |
| M200005601 |  |  |  |  |
| M200008541 | Jundm2 | NM_030887 | AB077438 | Jun dimerization protein 2 |
| M400005620 | Nfil3 | NM_017373 |  | nuclear factor, interleukin 3, regulated |
| **Posterior 0.5 Gy and 10.0 Gy** |  |  |  |  |
| M200012232 | Rad9 | NM_011237 | AF045663 | RAD9 homolog (S. pombe) |
| **Hind Limb 0.5 Gy and 2.0 Gy** |  |  |  |  |
| M200001988 | Sgne1 | NM_009162 |  | secretory granule neuroendocrine protein 1, 7B2 protein |
| M200003725 | Pscd3 | NM_011182 | BC035296 | pleckstrin homology, Sec7 and coiled-coil domains 3 |
| M200007299 | Slc41a3 | XM_132686 |  | PREDICTED: solute carrier family 41, member 3 |
| M400014572 |  |  |  |  |
| **Hind Limb 2.0 Gy and 10.0 Gy** |  |  |  |  |
| M200003339 | Sdcbp | NM_016807 |  | syndecan binding protein |
| M200003725 | Pscd3 | NM_011182 | BC035296 | pleckstrin homology, Sec7 and coiled-coil domains 3 |
| M200007299 | Slc41a3 | XM_132686 |  | PREDICTED: solute carrier family 41, member 3 |
| M200009937 |  | NM_025670 |  | RIKEN cDNA 5730403B10 gene (5730403B10Rik) |
| M200012232 | Rad9 | NM_011237 | AF045663 | RAD9 homolog (S. pombe) |
| M200014557 |  | XM_127336 |  | PREDICTED: SECIS binding protein 2 (Secisbp2) |
| M300021075 | Il31ra | NM_139299 | AB083111 | interleukin 31 receptor A |
| M400014572 |  |  |  |  |
| **Hind Limb 0.5 Gy and 10.0 Gy** |  |  |  |  |
| M200003725 | Pscd3 | NM_011182 | BC035296 | pleckstrin homology, Sec7 and coiled-coil domains 3 |
| M200007299 | Slc41a3 | XM_132686 |  | PREDICTED: solute carrier family 41, member 3 |
| M400014572 |  |  |  |  |
