# Supplementary material for: Diagnosis of Partial Body Radiation Exposure in Mice Using Peripheral Blood Gene Expression Profiles
Source: PLoS One. 2010 Jul 12;5(7):e11535. doi: 10.1371/journal.pone.0011535 (PMC2902517; doi:10.1371/journal.pone.0011535)
Supplement: Table S3 — Overlapping genes between TBI and Partial Body Signatures. (0.05 MB DOC) [file pone.0011535.s003.doc]

**Table S3. Overlapping genes between TBI and Partial Body Signatures**

| **Operon OligoID** | **Gene Symbol** | **RefSeq** | **GenBank** | **Description** |
| --- | --- | --- | --- | --- |
| **0.5 Gy Anterior vs 0.5 Gy TBI** |  |  |  |  |
| NONE |  |  |  |  |
| **2.0 Gy Anterior vs 2.0 Gy TBI** |  |  |  |  |
| NONE |  |  |  |  |
| **10.0 Gy Anterior vs 10.0 Gy TBI** |  |  |  |  |
| NONE |  |  |  |  |
| **0.5 Gy Posterior vs 0.5 Gy TBI** |  |  |  |  |
| NONE |  |  |  |  |
| **2.0 Gy Posterior vs 2.0 Gy TBI** |  |  |  |  |
| NONE |  |  |  |  |
| **10.0 Gy Posterior vs 10.0 Gy TBI** |  |  |  |  |
| M200007578 | Cdkn1a | NM_007669 |  | cyclin-dependent kinase inhibitor 1A (P21) |
| M200006364 | Dcxr | NM_026428 |  | dicarbonyl L-xylulose reductase |
| **0.5 Gy Hind Limb vs 0.5 Gy TBI** |  |  |  |  |
| M400006052 |  | XM_356705 |  | PREDICTED: similar to 60S ribosomal protein L19 (LOC382844) |
| **2.0 Gy Hind Limb vs 2.0 Gy TBI** |  |  |  |  |
| M200003339 | Sdcbp | NM_016807 |  | syndecan binding protein |
| **10.0 Gy Hind Limb vs 10.0 Gy TBI** |  |  |  |  |
| M200008116 |  | NM_028766 |  | RIKEN cDNA 1200015A22 gene (1200015A22Rik) |
| M200013566 | Gng12 | NM_025278 |  | guanine nucleotide binding protein (G protein), gamma 12 |
| M200003339 | Sdcbp | NM_016807 |  | syndecan binding protein |
